# Supplementary material for: Cross-Cell-Type Prediction of TF-Binding Site by Integrating Convolutional Neural Network and Adversarial Network
Source: Int J Mol Sci. 2019 Jul 12;20(14):3425. doi: 10.3390/ijms20143425 (PMC6679139; doi:10.3390/ijms20143425)
Supplement: Supplementary file 1 [file ijms-20-03425-s001.zip › Supplementary material/Supplementary material.docx]

Supplement to “Cross-cell-type prediction of TF-binding Site by Integrating Convolutional Neural Network and Adversarial Network”

Gongqiang Lan, Jiyun Zhou, Ruifeng Xu, Qin Lu, Hongpeng Wang

May 13, 2019

1. **Supplementary Tables**

Table S1. The impacts of two negative sequence generation methods on the performance of DANN_TF and the baseline method (Please refer to Table S1.xlsx)

Table S2. AUCs and F1 scores of data augmentation by DANN_TF and the baseline method

| TF | Cell  type | AUC  DANN_TF Baseline *p value* | | | *F*1  DANN_TF Baseline *p value* | | |
| --- | --- | --- | --- | --- | --- | --- | --- |
| CTCF | GM12878 | 0.9631 | **0.9646** | 7.13e-02 | 0.9250 | **0.9300** | 4.81e-03 |
|  | H1-hESC | **0.9657** | 0.9646 | 2.03e-03 | **0.9239** | 0.9211 | 9.72e-03 |
|  | HeLa-S3 | 0.9383 | **0.9399** | 2.83e-06 | 0.8959 | **0.8974** | 1.86e-02 |
|  | HepG2 | 0.9693 | **0.9705** | 1.64e-02 | **0.9360** | 0.9350 | 2.21e-01 |
|  | K562 | 0.9476 | **0.9494** | 3.64e-02 | **0.9113** | 0.9109 | 3.34e-01 |
| GABPA | GM12878 | **0.9797** | 0.9760 | 7.13e-02 | **0.9521** | 0.9385 | 1.85e-03 |
|  | H1-hESC | **0.9472** | 0.9448 | 2.03e-03 | **0.8721** | 0.8706 | 3.65e-03 |
|  | HeLa-S3 | **0.9692** | 0.9624 | 2.83e-06 | **0.9334** | 0.9246 | 8.87e-05 |
|  | HepG2 | **0.9842** | 0.9809 | 1.64e-02 | **0.9492** | 0.9396 | 4.33e-03 |
|  | K562 | **0.9594** | 0.9573 | 3.64e-02 | **0.9093** | 0.9029 | 6.93e-03 |
| JunD | GM12878 | **0.8249** | 0.7330 | 2.24e-06 | **0.6686** | 0.5057 | 3.45e-07 |
|  | H1-hESC | **0.9329** | 0.8890 | 1.31e-04 | **0.8371** | 0.8203 | 3.72e-04 |
|  | HeLa-S3 | **0.9581** | 0.9424 | 8.68e-09 | **0.9003** | 0.8871 | 1.00e-04 |
|  | HepG2 | **0.9733** | 0.9704 | 2.52e-06 | **0.9317** | 0.9282 | 7.14e-04 |
|  | K562 | **0.9376** | 0.9272 | 4.09e-11 | **0.8510** | 0.8260 | 5.01e-06 |
| REST | GM12878 | **0.9575** | 0.9403 | 2.12e-05 | **0.8961** | 0.8712 | 1.36e-06 |
|  | H1-hESC | **0.9780** | 0.9716 | 3.62e-04 | **0.9444** | 0.9325 | 1.55e-05 |
|  | HeLa-S3 | **0.9785** | 0.9750 | 1.03e-04 | **0.9481** | 0.9412 | 4.64e-03 |
|  | HepG2 | **0.9864** | 0.9851 | 3.49e-01 | **0.9595** | 0.9497 | 4.16e-02 |
|  | K562 | **0.9666** | 0.9639 | 5.79e-03 | **0.9282** | 0.9223 | 2.43e-03 |
| USF2 | GM12878 | **0.9741** | 0.9717 | 4.97e-01 | **0.9287** | 0.9236 | 7.89e-03 |
|  | H1-hESC | **0.9649** | 0.9469 | 2.82e-09 | **0.9114** | 0.8902 | 1.84e-04 |
|  | HeLa-S3 | **0.9628** | 0.9606 | 2.05e-03 | **0.9127** | 0.9041 | 2.47e-04 |
|  | HepG2 | **0.9689** | 0.9683 | 3.11e-01 | **0.9257** | 0.9184 | 1.17e-03 |
|  | K562 | **0.9767** | 0.9734 | 5.14e-02 | **0.9353** | 0.9296 | 1.43e-02 |

Table S3. AUCs and F1 scores of semi-supervised prediction by DANN_TF and the baseline method (50% target training data is labeled)

| TF | Cell  type | AUC  DANN_TF Baseline *p value* | | | *F*1  DANN_TF Baseline *p value* | | |
| --- | --- | --- | --- | --- | --- | --- | --- |
| CTCF | GM12878 | **0.9651** | 0.9648 | 4.06e-03 | **0.9327** | 0.9297 | 4.33e-03 |
|  | H1-hESC | **0.9667** | 0.9637 | 1.44e-06 | **0.9261** | 0.9182 | 5.95e-07 |
|  | HeLa-S3 | **0.9411** | 0.9397 | 4.54e-03 | **0.9017** | 0.8963 | 1.88e-03 |
|  | HepG2 | **0.9713** | 0.9704 | 3.43e-03 | **0.9383** | 0.9342 | 8.70e-04 |
|  | K562 | **0.9507** | 0.9491 | 3.31e-02 | **0.9162** | 0.9099 | 6.00e-06 |
| GABPA | GM12878 | **0.9819** | 0.9744 | 1.54e-04 | **0.9507** | 0.9364 | 5.56e-04 |
|  | H1-hESC | **0.9454** | 0.9393 | 2.55e-05 | **0.8698** | 0.8639 | 8.55e-04 |
|  | HeLa-S3 | **0.9682** | 0.9585 | 1.09e-06 | **0.9325** | 0.9204 | 5.10e-06 |
|  | HepG2 | **0.9837** | 0.9806 | 1.40e-02 | **0.9546** | 0.9388 | 1.57e-06 |
|  | K562 | **0.9597** | 0.9565 | 7.92e-02 | **0.9088** | 0.8982 | 2.60e-03 |
| JunD | GM12878 | **0.8154** | 0.7285 | 9.96e-08 | **0.6528** | 0.5296 | 1.52e-07 |
|  | H1-hESC | **0.9182** | 0.8672 | 1.34e-05 | **0.8311** | 0.8070 | 3.57e-03 |
|  | HeLa-S3 | **0.9547** | 0.9422 | 1.05e-06 | **0.8973** | 0.8862 | 2.93e-03 |
|  | HepG2 | **0.9705** | 0.9669 | 4.08e-05 | **0.9273** | 0.9250 | 1.86e-03 |
|  | K562 | **0.9305** | 0.9164 | 4.49e-13 | **0.8362** | 0.8165 | 1.15e-04 |
| REST | GM12878 | **0.9559** | 0.9367 | 7.90e-09 | **0.8986** | 0.8504 | 1.19e-07 |
|  | H1-hESC | **0.9770** | 0.9681 | 2.65e-06 | **0.9425** | 0.9254 | 1.99e-05 |
|  | HeLa-S3 | **0.9775** | 0.9728 | 8.87e-07 | **0.9475** | 0.9404 | 6.13e-05 |
|  | HepG2 | **0.9887** | 0.9847 | 2.60e-01 | **0.9623** | 0.9505 | 9.04e-03 |
|  | K562 | **0.9659** | 0.9604 | 1.44e-04 | **0.9257** | 0.9191 | 4.22e-03 |
| USF2 | GM12878 | **0.9733** | 0.9708 | 1.18e-02 | **0.9250** | 0.9216 | 5.49e-04 |
|  | H1-hESC | **0.9626** | 0.9385 | 1.83e-09 | **0.9068** | 0.8895 | 8.32e-03 |
|  | HeLa-S3 | **0.9628** | 0.9587 | 5.79e-07 | **0.9091** | 0.9026 | 1.80e-03 |
|  | HepG2 | **0.9699** | 0.9660 | 4.89e-05 | **0.9263** | 0.9148 | 1.04e-04 |
|  | K562 | **0.9751** | 0.9735 | 7.83e-02 | **0.9366** | 0.9326 | 6.37e-02 |

Table S4. AUCs and F1 scores of semi-supervised prediction by DANN_TF and the baseline method (20% target training data is labeled)

| TF | Cell  type | AUC  DANN_TF Baseline *p value* | | | *F*1  DANN_TF Baseline *p value* | | |
| --- | --- | --- | --- | --- | --- | --- | --- |
| CTCF | GM12878 | **0.9676** | 0.9644 | 3.68e-04 | **0.9369** | 0.9282 | 5.63e-05 |
|  | H1-hESC | **0.9665** | 0.9623 | 2.20e-07 | **0.9272** | 0.9172 | 1.72e-09 |
|  | HeLa-S3 | **0.9426** | 0.9400 | 3.93e-03 | **0.9047** | 0.8973 | 3.28e-06 |
|  | HepG2 | **0.9727** | 0.9698 | 2.74e-07 | **0.9430** | 0.9333 | 5.64e-11 |
|  | K562 | **0.9520** | 0.9487 | 3.52e-04 | **0.9199** | 0.9099 | 2.70e-08 |
| GABPA | GM12878 | **0.9817** | 0.9749 | 8.65e-03 | **0.9483** | 0.9344 | 3.28e-03 |
|  | H1-hESC | **0.9420** | 0.9365 | 3.16e-03 | **0.8675** | 0.8612 | 1.31e-03 |
|  | HeLa-S3 | **0.9653** | 0.9553 | 5.75e-06 | **0.9209** | 0.9185 | 1.37e-04 |
|  | HepG2 | **0.9836** | 0.9802 | 1.50e-02 | **0.9495** | 0.9363 | 2.75e-05 |
|  | K562 | **0.9583** | 0.9554 | 1.28e-02 | **0.9043** | 0.8929 | 3.73e-04 |
| JunD | GM12878 | **0.8065** | 0.7194 | 5.78e-06 | **0.6426** | 0.5380 | 2.25e-05 |
|  | H1-hESC | **0.8882** | 0.8463 | 8.80e-06 | **0.8188** | 0.7690 | 1.58e-06 |
|  | HeLa-S3 | **0.9506** | 0.9414 | 3.81e-05 | **0.8935** | 0.8847 | 1.52e-03 |
|  | HepG2 | **0.9663** | 0.9633 | 2.28e-04 | **0.9197** | 0.9181 | 1.39e-02 |
|  | K562 | **0.9188** | 0.9073 | 8.79e-11 | **0.8167** | 0.8080 | 4.10e-03 |
| REST | GM12878 | **0.9563** | 0.9307 | 8.03e-09 | **0.8917** | 0.8437 | 1.12e-06 |
|  | H1-hESC | **0.9756** | 0.9674 | 3.29e-05 | **0.9422** | 0.9255 | 8.25e-07 |
|  | HeLa-S3 | **0.9767** | 0.9736 | 4.50e-03 | **0.9486** | 0.9384 | 2.75e-04 |
|  | HepG2 | **0.9882** | 0.9841 | 1.24e-01 | **0.9638** | 0.9404 | 1.08e-04 |
|  | K562 | **0.9648** | 0.9593 | 7.47e-06 | **0.9214** | 0.9114 | 6.73e-03 |
| USF2 | GM12878 | **0.9717** | 0.9698 | 4.25e-03 | **0.9296** | 0.9194 | 9.54e-03 |
|  | H1-hESC | **0.9608** | 0.9332 | 4.04e-08 | **0.9060** | 0.8791 | 1.04e-03 |
|  | HeLa-S3 | **0.9595** | 0.9562 | 9.46e-05 | **0.9049** | 0.8978 | 3.72e-03 |
|  | HepG2 | **0.9693** | 0.9652 | 9.57e-05 | **0.9228** | 0.9123 | 1.72e-03 |
|  | K562 | **0.9756** | 0.9723 | 8.39e-04 | **0.9352** | 0.9292 | 5.46e-04 |

Table S5. AUCs and F1 scores of semi-supervised prediction by DANN_TF and the baseline method (10% target training data is labeled)

| TF | Cell  type | AUC  DANN_TF Baseline *p value* | | | *F*1  DANN_TF Baseline *p value* | | |
| --- | --- | --- | --- | --- | --- | --- | --- |
| CTCF | GM12878 | **0.9670** | 0.9645 | 4.56e-04 | **0.9382** | 0.9277 | 2.10e-07 |
|  | H1-hESC | **0.9655** | 0.9618 | 7.51e-07 | **0.9255** | 0.9158 | 4.01e-09 |
|  | HeLa-S3 | **0.9429** | 0.9394 | 1.07e-03 | **0.9053** | 0.8963 | 1.96e-07 |
|  | HepG2 | **0.9729** | 0.9701 | 2.10e-06 | **0.9426** | 0.9334 | 4.26e-06 |
|  | K562 | **0.9523** | 0.9490 | 4.01e-04 | **0.9185** | 0.9095 | 7.92e-07 |
| GABPA | GM12878 | **0.9816** | 0.9759 | 6.21e-03 | **0.9472** | 0.9337 | 4.56e-03 |
|  | H1-hESC | **0.9398** | 0.9340 | 5.59e-03 | **0.8677** | 0.8578 | 2.38e-03 |
|  | HeLa-S3 | **0.9603** | 0.9546 | 1.32e-02 | **0.9209** | 0.9185 | 2.60e-01 |
|  | HepG2 | **0.9827** | 0.9804 | 6.58e-02 | **0.9483** | 0.9348 | 5.53e-04 |
|  | K562 | **0.9591** | 0.9556 | 5.61e-02 | **0.9086** | 0.8929 | 5.50e-03 |
| JunD | GM12878 | **0.7959** | 0.7093 | 1.69e-05 | **0.6426** | 0.5362 | 6.02e-06 |
|  | H1-hESC | **0.8689** | 0.8330 | 2.13e-09 | **0.8021** | 0.7433 | 1.26e-04 |
|  | HeLa-S3 | **0.9478** | 0.9398 | 3.19e-06 | **0.8936** | 0.8812 | 9.14e-03 |
|  | HepG2 | **0.9632** | 0.9617 | 2.24e-03 | **0.9169** | 0.9146 | 3.02e-01 |
|  | K562 | **0.9126** | 0.9031 | 8.51e-08 | **0.8103** | 0.8022 | 1.87e-03 |
| REST | GM12878 | **0.9521** | 0.9299 | 8.38e-09 | **0.8866** | 0.8345 | 2.72e-06 |
|  | H1-hESC | **0.9746** | 0.9660 | 7.70e-06 | **0.9384** | 0.9225 | 3.67e-06 |
|  | HeLa-S3 | **0.9764** | 0.9719 | 6.59e-05 | **0.9460** | 0.9369 | 5.03e-04 |
|  | HepG2 | **0.9877** | 0.9835 | 6.67e-02 | **0.9577** | 0.9462 | 8.65e-03 |
|  | K562 | **0.9617** | 0.9564 | 2.79e-04 | **0.9182** | 0.9111 | 7.02e-04 |
| USF2 | GM12878 | **0.9713** | 0.9694 | 1.07e-03 | **0.9271** | 0.9202 | 9.38e-03 |
|  | H1-hESC | **0.9587** | 0.9326 | 1.71e-05 | **0.8968** | 0.8735 | 1.04e-03 |
|  | HeLa-S3 | **0.9587** | 0.9556 | 1.21e-04 | **0.9047** | 0.8991 | 2.04e-03 |
|  | HepG2 | **0.9688** | 0.9649 | 2.09e-04 | **0.9224** | 0.9116 | 6.03e-04 |
|  | K562 | **0.9751** | 0.9724 | 4.82e-03 | **0.9353** | 0.9276 | 1.00e-04 |

Table S6. AUCs and F1 scores of cross-cell-type prediction by DANN_TF and the baseline method

| TF | Cell  type | AUC  DANN_TF Baseline *p value* | | | *F*1  DANN_TF Baseline *p value* | | |
| --- | --- | --- | --- | --- | --- | --- | --- |
| CTCF | GM12878 | **0.9673** | 0.9640 | 5.63e-05 | **0.9378** | 0.9281 | 1.08e-06 |
|  | H1-hESC | **0.9646** | 0.9622 | 1.40e-05 | **0.9260** | 0.9161 | 6.02e-08 |
|  | HeLa-S3 | **0.9433** | 0.9396 | 3.82e-05 | **0.9066** | 0.8970 | 4.69e-07 |
|  | HepG2 | **0.9730** | 0.9701 | 3.22e-06 | **0.9418** | 0.9324 | 2.43e-07 |
|  | K562 | **0.9526** | 0.9491 | 1.07e-03 | **0.9184** | 0.9084 | 1.00e-08 |
| GABPA | GM12878 | **0.9796** | 0.9742 | 1.48e-03 | **0.9444** | 0.9324 | 1.29e-03 |
|  | H1-hESC | **0.9398** | 0.9336 | 7.05e-03 | **0.8690** | 0.8578 | 1.28e-03 |
|  | HeLa-S3 | **0.9553** | 0.9544 | 3.81e-01 | **0.9148** | 0.9155 | 4.35e-01 |
|  | HepG2 | **0.9827** | 0.9805 | 6.99e-02 | **0.9476** | 0.9377 | 5.60e-04 |
|  | K562 | **0.9568** | 0.9555 | 1.33e-02 | **0.9082** | 0.8929 | 1.86e-04 |
| JunD | GM12878 | **0.7883** | 0.7039 | 1.91e-05 | **0.6275** | 0.5330 | 8.44e-05 |
|  | H1-hESC | **0.8286** | 0.8187 | 1.76e-07 | **0.7270** | 0.7166 | 1.18e-04 |
|  | HeLa-S3 | **0.9430** | 0.9380 | 4.30e-03 | **0.8893** | 0.8825 | 1.25e-03 |
|  | HepG2 | **0.9630** | 0.9600 | 2.98e-05 | **0.9118** | 0.9017 | 9.80e-09 |
|  | K562 | **0.9046** | 0.8989 | 5.90e-06 | **0.8083** | 0.7992 | 1.21e-02 |
| REST | GM12878 | **0.9332** | 0.9221 | 2.58e-03 | **0.8430** | 0.8269 | 1.07e-03 |
|  | H1-hESC | **0.9723** | 0.9652 | 3.05e-04 | **0.9357** | 0.9202 | 1.92e-04 |
|  | HeLa-S3 | **0.9759** | 0.9717 | 2.10e-04 | **0.9466** | 0.9385 | 2.17e-03 |
|  | HepG2 | **0.9866** | 0.9838 | 2.21e-01 | **0.9535** | 0.9465 | 1.41e-01 |
|  | K562 | **0.9595** | 0.9549 | 9.01e-04 | **0.9174** | 0.9093 | 1.08e-03 |
| USF2 | GM12878 | **0.9708** | 0.9694 | 3.45e-03 | **0.9233** | 0.9183 | 6.56e-03 |
|  | H1-hESC | **0.9577** | 0.9360 | 1.57e-05 | **0.8950** | 0.8727 | 4.17e-06 |
|  | HeLa-S3 | **0.9571** | 0.9549 | 9.96e-03 | **0.9017** | 0.8950 | 9.15e-04 |
|  | HepG2 | **0.9677** | 0.9648 | 2.29e-03 | **0.9184** | 0.9105 | 9.94e-03 |
|  | K562 | **0.9741** | 0.9721 | 3.99e-03 | **0.9328** | 0.9290 | 7.84e-03 |

Table S7. Details of AUC and F1 score of cross-cell-type prediction by DANN_TF and the baseline method and supervised prediction by the baseline method on the additional 13 TFs in the five cell-types. (Please refer to Table S7.xlsx)

Table S8. Details of F1 score comparison between DANN_TF and the baseline method for cross-cell-type prediction.

| Cell-type | GM12878 | | H1-hESC | HeLa-S3 | HepG2 | K562 | Average^b^ | |
| --- | --- | --- | --- | --- | --- | --- | --- | --- |
| Sample total | | 13 | 13 | 13 | 13 | 13 | | 13 |
| Improvement total | | 11 | 11 | 12 | 11 | 11 | | 11.2 |
| Improvement (%) | | 84.6 | 84.6 | 92.3 | 84.6 | 84.6 | | 86.2 |
| Maximum (%) | | 3.8 | 3.0 | 7.6 | 3.1 | 7.9 | | 5.08 |
| Average^a^ (%) | | 1.6 | 1.2 | 2.8 | 1.5 | 2.1 | | 1.84 |

^a^ denotes the average improvement, ^c^ denotes the micro average over the total number of samples.

Table S9. Details of F1 score comparison between cross-cell-type prediction by DANN_TF and supervised prediction by the baseline method.

| Cell-type | GM12878 | | H1-hESC | HeLa-S3 | HepG2 | K562 | Average^b^ | |
| --- | --- | --- | --- | --- | --- | --- | --- | --- |
| Sample total | | 13 | 13 | 13 | 13 | 13 | | 13 |
| Improvement total | | 12 | 12 | 10 | 11 | 13 | | 11.6 |
| Improvement (%) | | 92.3 | 92.3 | 76.9 | 84.6 | 100 | | 89.2 |
| Maximum (%) | | 16.5 | 24.3 | 15.3 | 17.3 | 18.8 | | 18.44 |
| Average^a^ (%) | | 4.8 | 7.7 | 3.7 | 6.3 | 7.8 | | 6.06 |

^a^ denotes the average improvement, ^c^ denotes the micro average over the total number of samples.

Table S10. AUC performance comparison among data augmentation, semi-supervised and cross-cell-type prediction by DANN_TF

| TF | Cell  type | AUC | | | | |
| --- | --- | --- | --- | --- | --- | --- |
|  |  | data augment-  tation | semi-  supervised  (50%) | semi-  supervised  (20%) | semi-  supervised  (10%) | cross-  cell-type |
| CTCF | GM12878 | 0.9631 | 0.9651 | **0.9676** | 0.9670 | 0.9673 |
|  | H1-hESC | 0.9657 | **0.9667** | 0.9665 | 0.9655 | 0.9646 |
|  | HeLa-S3 | 0.9383 | 0.9411 | 0.9426 | 0.9429 | **0.9433** |
|  | HepG2 | 0.9693 | 0.9713 | 0.9727 | 0.9729 | **0.9730** |
|  | K562 | 0.9476 | 0.9507 | 0.9520 | 0.9523 | **0.9526** |
| GABPA | GM12878 | 0.9797 | **0.9819** | 0.9817 | 0.9816 | 0.9796 |
|  | H1-hESC | **0.9472** | 0.9454 | 0.9420 | 0.9398 | 0.9398 |
|  | HeLa-S3 | **0.9692** | 0.9682 | 0.9653 | 0.9603 | 0.9553 |
|  | HepG2 | **0.9842** | 0.9837 | 0.9836 | 0.9827 | 0.9827 |
|  | K562 | 0.9594 | **0.9597** | 0.9583 | 0.9591 | 0.9568 |
| JunD | GM12878 | **0.8249** | 0.8154 | 0.8065 | 0.7959 | 0.7883 |
|  | H1-hESC | **0.9329** | 0.9182 | 0.8882 | 0.8689 | 0.8286 |
|  | HeLa-S3 | **0.9581** | 0.9547 | 0.9506 | 0.9478 | 0.9430 |
|  | HepG2 | **0.9733** | 0.9705 | 0.9663 | 0.9632 | 0.9630 |
|  | K562 | **0.9376** | 0.9305 | 0.9188 | 0.9126 | 0.9046 |
| REST | GM12878 | **0.9575** | 0.9559 | 0.9563 | 0.9521 | 0.9332 |
|  | H1-hESC | **0.9780** | 0.9770 | 0.9756 | 0.9746 | 0.9723 |
|  | HeLa-S3 | **0.9785** | 0.9775 | 0.9767 | 0.9764 | 0.9759 |
|  | HepG2 | 0.9864 | **0.9887** | 0.9882 | 0.9877 | 0.9866 |
|  | K562 | **0.9666** | 0.9659 | 0.9648 | 0.9617 | 0.9595 |
| USF2 | GM12878 | **0.9741** | 0.9733 | 0.9717 | 0.9713 | 0.9708 |
|  | H1-hESC | **0.9649** | 0.9626 | 0.9608 | 0.9587 | 0.9577 |
|  | HeLa-S3 | **0.9628** | **0.9628** | 0.9595 | 0.9587 | 0.9571 |
|  | HepG2 | 0.9689 | **0.9699** | 0.9693 | 0.9688 | 0.9677 |
|  | K562 | **0.9767** | 0.9751 | 0.9756 | 0.9751 | 0.9741 |

Table S11. F1 score comparison among data augmentation, semi-supervised and cross-cell-type prediction by DANN_TF

| TF | Cell  type | F1 | | | | |
| --- | --- | --- | --- | --- | --- | --- |
|  |  | data augment-  tation | semi-  supervised  (50%) | semi-  supervised  (20%) | semi-  supervised  (10%) | cross-  cell-type |
| CTCF | GM12878 | 0.9250 | 0.9327 | 0.9369 | **0.9382** | 0.9378 |
|  | H1-hESC | 0.9239 | 0.9261 | **0.9272** | 0.9255 | 0.9260 |
|  | HeLa-S3 | 0.8959 | 0.9017 | 0.9047 | 0.9053 | **0.9066** |
|  | HepG2 | 0.9360 | 0.9383 | **0.9430** | 0.9426 | 0.9418 |
|  | K562 | 0.9113 | 0.9162 | **0.9199** | 0.9185 | 0.9184 |
| GABPA | GM12878 | **0.9521** | 0.9507 | 0.9483 | 0.9472 | 0.9444 |
|  | H1-hESC | **0.8721** | 0.8698 | 0.8675 | 0.8677 | 0.8690 |
|  | HeLa-S3 | **0.9334** | 0.9325 | 0.9209 | 0.9209 | 0.9148 |
|  | HepG2 | 0.9492 | **0.9546** | 0.9495 | 0.9483 | 0.9476 |
|  | K562 | **0.9093** | 0.9088 | 0.9043 | 0.9086 | 0.9082 |
| JunD | GM12878 | **0.6686** | 0.6528 | 0.6426 | 0.6426 | 0.6275 |
|  | H1-hESC | **0.8371** | 0.8311 | 0.8188 | 0.8021 | 0.7270 |
|  | HeLa-S3 | **0.9003** | 0.8973 | 0.8935 | 0.8936 | 0.8893 |
|  | HepG2 | **0.9317** | 0.9273 | 0.9197 | 0.9169 | 0.9118 |
|  | K562 | **0.8510** | 0.8362 | 0.8167 | 0.8103 | 0.8083 |
| REST | GM12878 | 0.8961 | **0.8986** | 0.8917 | 0.8866 | 0.8430 |
|  | H1-hESC | **0.9444** | 0.9425 | 0.9422 | 0.9384 | 0.9357 |
|  | HeLa-S3 | 0.9481 | 0.9475 | **0.9486** | 0.9460 | 0.9466 |
|  | HepG2 | 0.9595 | 0.9623 | **0.9638** | 0.9577 | 0.9535 |
|  | K562 | **0.9282** | 0.9257 | 0.9214 | 0.9182 | 0.9174 |
| USF2 | GM12878 | 0.9287 | 0.9250 | **0.9296** | 0.9271 | 0.9233 |
|  | H1-hESC | **0.9114** | 0.9068 | 0.9060 | 0.8968 | 0.8950 |
|  | HeLa-S3 | **0.9127** | 0.9091 | 0.9049 | 0.9047 | 0.9017 |
|  | HepG2 | 0.9257 | **0.9263** | 0.9228 | 0.9224 | 0.9184 |
|  | K562 | 0.9353 | **0.9366** | 0.9352 | 0.9353 | 0.9328 |


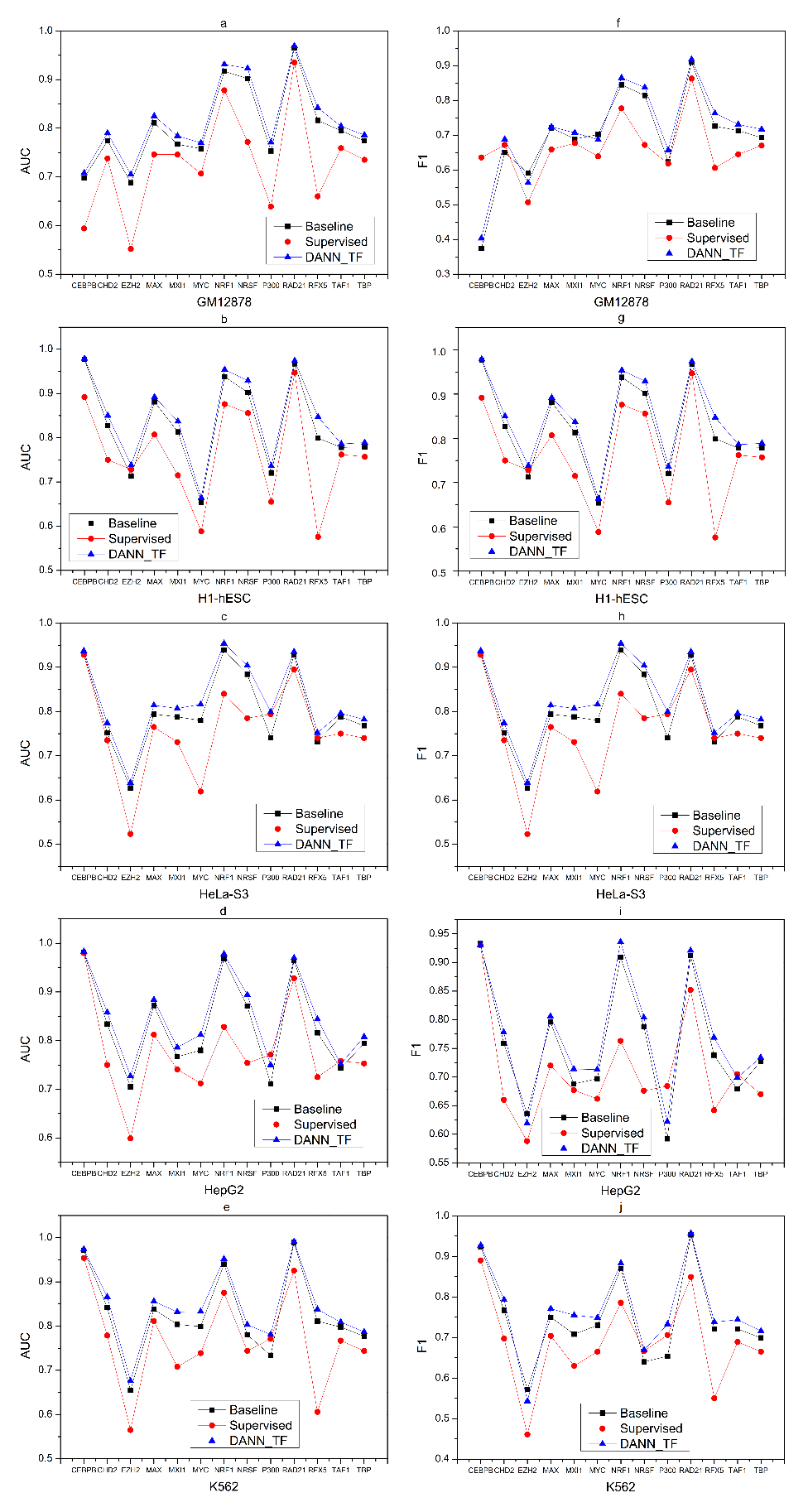


Figure S1 (a) - (e): AUC performance of cross-cell-type prediction by DANN_TF and the baseline method and supervised prediction by the baseline method for the 13 TFs in the five cell-types. (f) - (j): F1 score of cross-cell-type prediction by DANN_TF and the baseline method and supervised prediction by the baseline method for the 13 TFs in the five cell-types.
